# Does inpatient health services utilization vary by remoteness in the medical financial assistance population? Evidence from Shaanxi province, China

**DOI:** 10.1186/s12913-020-05907-x

**Published:** 2020-11-19

**Authors:** Yangling Ren, Zhongliang Zhou, Guanping Liu, Chi Shen, Dan Cao, Tiange Xu, Jane M. Fry, Rashed Nawaz, Dantong Zhao, Min Su, Tingshuai Ge, Yafei Si, Gang Chen

**Affiliations:** 1 School of Public Policy and Administration, Xi’an Jiaotong University, No. 28 Xianning West Road, Xi’an, 710049 Shaanxi China; 2 Centre for Health Economics, Monash University, 900 Dandenong Road, Caulfield East, VIC 3145 Australia; 3 School of Public Administration, Inner Mongolia University, Inner Mongolia, 010021 China; 4 ARC Centre of Excellence in Population Ageing Research (CEPAR), The University of New South Wales, 223 Anzac Parade, Sydney, NSW 2052 Australia

**Keywords:** Medical financial assistance (MFA), Geographic access, Inpatient care use, Moderation, China

## Abstract

**Background:**

Medical Financial Assistance (MFA) provides health insurance and financial support for millions of low income and disabled Chinese people, yet there has been little systematic analysis focused on this vulnerable population. This study aims to advance our understanding of MFA recipients’ access to health care and whether their inpatient care use varies by remoteness.

**Methods:**

Data were collected from the Surveillance System of Civil Affairs of Shaanxi province in 2016. To better proxy remoteness (geographic access), drive time from the respondent’s village to the nearest county-level or city-level hospital was obtained by a web crawler. Multilevel models were used to explore the impacts of remoteness on inpatient services utilization by MFA recipients. Furthermore, the potential moderating role of hospital grade (i.e. the grade of medical institution where recipient’s latest inpatient care services were taken in the previous year) on the relationship between geographic access and inpatient care use was explored.

**Results:**

The analytical sample consisted of 9516 inpatient claims within 73 counties of Shaanxi province in 2016. We find that drive time to the nearest hospital and hospital grade are salient predictors of inpatient care use and there is a significant moderation effect of hospital grade. Compared to those with shortest drive time to the nearest hospital, longer drive time is associated with a longer inpatient stay but fewer admissions and lower annual total and out-of-pocket (OOP) inpatient costs. In addition, these associations are lower when recipients are admitted to a tertiary hospital, for annual total and OOP inpatient expenditures, but higher for length of the most recent inpatient stay no matter what medical treatments are taken in secondary or tertiary hospitals for the most remote recipients.

**Conclusion:**

Our results suggest that remoteness has a significant and negative association with the frequency of inpatient care use. These findings advance our understanding of inpatient care use of the extremely poor and provide meaningful insights for further MFA program development as well as pro-poor health strategies.

**Supplementary Information:**

The online version contains supplementary material available at 10.1186/s12913-020-05907-x.

## Background

Health is a prerequisite for an individual’s all-round development. The current health reform in China aims to enable residents to have equal access to quality and affordable essential health care. Substantial progress has been achieved in ameliorating health care access including the establishment of basic medical insurance schemes (e.g. the New Cooperative Medical Scheme in rural areas and the Urban Resident Basic Medical Insurance in urban areas) that cover all residents, yet there is very limited evidence for the most financially disadvantaged population [1, 2]. According to the statistics from the National Poverty Alleviation and Development Information System, illness remained the top cause of poverty in rural poor households. Among more than 70 million poor families in China, the proportion of poverty caused by illness increased from 42% in 2013 to 44% in 2016 [3]. According to the Poverty Monitoring Report of rural China, in rural areas, the percentage of residents unable to receive timely health care due to financial handicaps dropped by 7.8%; however, the number increased by 10.1% due to long and essential drive times and transportation costs [4]. These vulnerable populations normally have poor health and live in rural and remote areas with limited access to health care [5–9]; they are also more likely to delay or deter essential medical treatments [10–12].

To protect the health of the vulnerable population and to reduce poverty, the Medical Financial Assistance (MFA) scheme was introduced in China as a purely pro-poor strategy. The MFA scheme targets extremely vulnerable individuals and families, and subsidizes basic medical insurance or provides cash assistance for medical care. In 2018, approximately 130 million individuals benefited from MFA [13]. A summary of eligibility criteria, basic information, and benefit packages of MFA is shown in Table 1. Although the MFA aims to expand basic medical insurance coverage, increase health care access, and to reduce financial risk for the poor, recent studies have found it has limited impacts on health promotion or financial risk protection, particularly for those who live in rural or remote areas [12, 14]. Furthermore, indirect costs, such as transportation costs, are not covered. Significant differences in health care access and use between rural and urban residents (geographic disparity) has made the limited access and delivery of health services for rural residents a widespread concern in China [5, 6, 15, 16].

**Table 1:** Summary of Medical Financial Assistance Scheme (MFA) at the national and provincial level

MFA	China (National level)	Shaanxi Province (Provincial level)
Basic information		
Implementation year	Piloted in rural areas in 2003 and urban areas in 2005; Implemented nationwide in 2008; Legitimized as an essential part of social assistance programs in 2014; Integrated rural and urban MFA in 2015	Implemented in 2012
Administration	Ministry of Civil Affairs of China	Originally administered by Ministry of Civil Affairs of Shaanxi Province, transferred to Shaanxi Provincial Healthcare Security Administration in 2020 because of institutional reform
Target population	1) Key recipients: “Dibao” (households enrolled in the Minimum Living Standard Scheme, the criterion is adjusted yearly and in 2020 for rural areas is household income less than 5336 RMB per year) and “Tekun” (the extremely poor households identified by the Draft Decree on Social Assistance); 2) Low-income recipients: identified by local government, the criterion is usually a monthly family income of between 100% and 120–150% of the local Minimum Living Standard line; “Wubao” (rural residents enrolled in the Five Guarantee Program) and individuals identified by county or above government; 3) Recipients who became poor due to illness: major illness imposes a large economic burden on the recipient, as well as a dilemma of maintaining basic life.	1) Key recipients: “Dibao” (households enrolled in the Minimum Living Standard Scheme, the criterion in 2018 for rural areas is household income less than 3470 RMB per year) and “Tekun” (the extremely poor households identified by the Draft Decree on Social Assistance); 2) Low-income recipients: identified by local government, the criterion is household income per capita is lower than 1.5 times the minimum living standard; severely disabled and elderly living around the Minimum Living Standard line;“Wubao” (rural residents enrolled in Five Guarantee Program) and needy residents identified by county or city government; 3) Recipients who became poor due to illness: major illness strike imposes a large economic burden on the recipient, as well as dilemma of maintaining basic life. 4) Specific recipients: “Youfu” (Regulations on Special Care and Treatment for Servicemen) and individuals who get injured in helping others.
Assistance method	1) Subsidizing health insurance schemes: subsidizing those targets to be enrolled in SHI ^a^, usually the New Cooperative Medical Scheme for rural residents and the Urban Resident Basic Medical Insurance for urban residents; 2) After medical treatment assistance: apply for cash assistance of MFA after using health care and paying medical expenses.	1) Subsidizing health insurance schemes: subsidizing those targets to be enrolled in SHI, usually the New Cooperative Medical Scheme for rural residents and the Urban Resident Basic Medical Insurance for urban residents; 2) Immediate assistance: receive health care at MFA designated medical institutions, carry valid medical documents at the hospital window to obtain MFA certification, and get compensation at once with recipients only needing to pay the remaining out-of-pocket (OOP) expenses. 3) After medical treatment assistance: apply to the government of the township and district officer for cash assistance of MFA after using health care and paying medical expenses by themselves.
Assisted population	87,204,000 person-times in 2016	437,508 person-times in 2016
Risk-pooling	County level	County level
Benefit packages		
Premiums	1) For enrollees participating in the SHI ^a^, there are no additional premiums for the MFA; 2) For residents without any health insurance, the premiums for the MFA are determined by local governments; or they can choose to participate in one SHI, usually the New Cooperative Medical Scheme for rural residents and the Urban Resident Basic Medical Insurance for urban residents, and premiums were partly or fully subsided by local government.	1) For enrollees participating in the basic health SHI, there is no additional premium for the MFA; 2) For residents without any health insurance, the premiums for the MFA are being county-specific, for example, 150 RMB per year for “Dibao” recipients in some counties; or they can choose to participate in one health insurance scheme, usually the New Cooperative Medical Scheme for rural residents and the Urban Resident Basic Medical Insurance for urban residents, and premiums were partly or fully subsided by local governments.
Reimbursement for outpatient care		
Ceiling of reimbursement	Not clearly unified on the national scale, leave it to the county or above government to determine	1) For daily outpatient services: not exceed 1000 RMB; 2) For the outpatient services of serious diseases: not exceed 5000 RMB;
Deductibles	Not clearly unified on the national scale, leave it to the county or above government to determine	1) Key recipients: no less than 50% for “Dibao” recipients, 100% for “Tekun” recipients; 2) Low-income recipients and specific recipients: determined by the county or above government.
Reimbursement for inpatient care		
Ceiling of reimbursement	Not clearly unified on the national scale, leave it to the county or above government to determine; 30,000 RMB to 50,000 RMB in most provinces, more than 80,000 RMB in cities like Beijing, Shanghai and Chongqing.	1) For basic inpatient services: reimburse all OOP expenses for “Tekun” recipients, not exceed 15,000 RMB for “Dibao” recipients, not exceed 12,000 RMB for low-income recipients; 2) For the inpatient services of serious diseases: reimburse all OOP expenses for “Tekun” recipients, not exceed 30,000 RMB for “Dibao” recipients, not exceed 20,000 RMB for low-income recipients, not exceed 15,000 RMB for recipients become poor due to illness.
Deductibles	Not less than 70%	1) Key recipients: not less than 70%; 2) Low-income recipients and specific recipients: not less than 50%.
Source of financing	1) Funded by Central government budget; lottery welfare fund; and donations from society and individuals. 2) Managed, appropriated and being accountable by county governments.	1) Funded by Central government subsides; Provincial, city and county government budgets; Provincial, city and county level lottery welfare funds; social donations; and interest income of special and independent MFA account; 2) Managed, appropriated and being accountable by county governments.

Note: Data were from National Healthcare Security Administration; National Health Statistical Yearbook; Ministry of Civil Affairs of China; Ministry of Civil Affairs of Shaanxi Province; Liu K et al [8]

^a^ Social health insurance schemes (SHI) including the New Cooperative Medical Scheme, the Urban Resident Basic Medical Insurance and the Urban Employee Basic Medical Insurance

Andersen’s Behavioral Model of Health Services Use (consisting of predisposing factors, enabling factors, and needs) has been widely used to study health care access [17–20]. Studies have also suggested that access to health care comprises financial access (medical costs and people’s capacity to pay) and geographic access [21]. Geographic access denotes essential distance or time to medical institutions; the distance that must be traveled (or travel time) to the health facility to receive health care limits access to health services. In China, studies of financial access to health care are well established but only a few focused on geographic access [8, 22]. They found that geographic access is one of the dominant factors hindering health services utilization in China, especially in rural areas of the western region, due to the long distance to medical institutions and the undersupply of transport services [23].

There is no evidence on whether geographic access influences the MFA population in China. This study aims to fill the gap by investigating whether inpatient services utilization varies by remoteness in MFA recipients. Moreover, given the important role played by the hospital grade in disparities of health services access and utilization in China [22, 24, 25], this study explores whether hospital grade acts as a moderator in the associations between geographic access and inpatient services utilization. The findings will advance our understanding of inpatient care use by the most vulnerable subgroups in China, as well as provide unique insights for future MFA program design and implementation.

## Methods

### Data source

The study site is Shaanxi Province, which is located in the northwest of China, with an annual per capita net income of a rural resident of 9396 RMB (Chinese Renminbi Yuan, 1 RMB = 0.14 US dollars) and ranked 26 among 31 provinces in China in 2016. In this region, 80% are mountainous or plateau areas, particularly in rural areas. In this study, community-level indicators were drawn from the National Bureau of Statistics 2017 and Shaanxi Health and Family Planning Statistical Yearbook 2017, while data on individual-level characteristics were obtained from the Surveillance System of Civil Affairs of Shaanxi Province in 2016. The Surveillance System was established in 2012, and includes the vulnerable populations who are eligible for the MFA in all 73 counties in Shaanxi Province. The Civil Affairs Departments are responsible for data collection and data, which is submitted annually.

The MFA data comprises three independent groups according to how assistance is received: in the first group, recipients receive subsidies for enrolling in basic medical insurance schemes; recipients from the second group receive immediate assistance during health service utilization, and the third group receive assistance only after medical treatment. The MFA data contains individual information, including personal demographics (e.g. name, gender, age, marital status, health status), household information (e.g. household income, family size, and residential address) and assistance details (e.g. reimbursement method; outpatient/inpatient medical costs: total cost, self-paid payment and reimbursement cost; and inpatient admission and discharge dates).

### Study sample

This study focuses on rural residents as they are the most vulnerable population. For the first two MFA groups introduced above, there was not sufficient personal information (e.g. economic status and complete hospitalization details) available to the researchers. Consequently, we focus on the third group, i.e. those who receive assistant after medical treatments. From the total of 11,346 recipients’ records in this group, we firstly exclude around 7% (*n* = 794) of records in which the inpatient care services were taken in township health centers which are normally located close to home and treat minor disease. Next, 1036 records with key information missing (e.g. inpatient medical costs) were dropped, leaving a final study sample of 9516 recipients within 73 counties.

### Empirical strategy

#### Geographic access

Travel barriers are represented by either straight-line distance, driving distance, or drive time to the nearest health institution and are frequently used to measure geographic access to health care [26]. Straight-line distance is intuitive and easily calculated, while actual driving distance and time are regarded as better alternatives as they factor in the actual road network likely to be used [27]. These indicators used to be difficult to measure but have now become much easier due to the advancement of web crawler techniques [22]. A web crawler is a search engine that can systematically visit web sites and collects the relevant information with high accuracy [22, 27, 28]. Here we use this technology to obtain the actual driving distances and time from a resident’s home to the nearest secondary/tertiary hospital in Shaanxi Province; all requests to the API (Application Program Interface) of Amap (China’s leading solution provider of digital map content, navigation and location services) were completed by the web crawler with a self-compiled Python 3.6 program.

The names of village clinics/neighborhood committees, county-level and city-level hospitals in Shaanxi province were obtained through the Shaanxi Provincial Health Statistical Annual Report and Shaanxi Rural Health Statistical Yearbook. Coordinates of the village clinics/neighborhood committees were taken as the starting points while the coordinates of the county/city hospitals were regarded as the terminal points and the path planning interface was used to collect navigation data. The data strategy chose the fastest route without taking highways to obtain the time and distance from the village clinics/neighborhood committees to the county-level/city-level hospitals. Taking account of traffic conditions at different times, four crawler requests for Amap were conducted on Friday, November 23, 2018 (from 10:00 am to 11:00 am) and Tuesday, November 27, 2018 (from 14:00 to 15:00 PM), and the average of four times was used.

Eventually, the data for 10,350 clinics/neighborhood committees were obtained from 13,074 clinics/neighborhood committees in 73 counties of Shaanxi province. Given the importance of drive time in medical treatment, particularly for emergency treatment and severe diseases, we used drive time from residential districts to the nearest secondary/tertiary hospital to measure remoteness [25, 27]. Drive time (in minutes) was categorized in five groups: the shortest (time < 30) = 1; shorter (30 < = time < 60) = 2; medium (60 < = time < 90) = 3; longer (90 < = time < 120) = 4; longest (time > 120) = 5. Without a unified and standard measurement for changes in driving time, we chose a 30-min increase as the cutoff point based on the data distribution and previous literature which suggests 30 min is a significant increase in driving time [29].

#### Outcome indicators

The primary outcome variables in this analysis are: (1) length of the latest inpatient stay last year, (2) number of admissions last year, (3) total inpatient expenditure, and (4) out-of-pocket (OOP) inpatient expenditure. Length of the latest inpatient stay is calculated as the number of days for latest hospitalization in the prior year, number of admissions last year is measured by the number of admissions during the previous year, while total inpatient expenditure and OOP inpatient expenditure are gauged using all the inpatient care costs and self-paid inpatient care costs in the past year, respectively.

#### Covariates

Our covariates were selected according to the framework of Anderson’s Behavioral Model: socio-demographic characteristics, e.g. gender, age, marital status, and education level were specified as predisposing factors; the presence of any chronic diseases and self-reported health status were selected as need factors. In terms of enabling factors that may facilitate health behaviors, economic status, driving time to the nearest hospital, and the hospital grade were grouped into individual-level enabling factors. Over the past decades, tremendous attention has been given to individual-level factors in examining their effects on health service utilization, while there is a growing appreciation that factors beyond individual characteristics also play important roles in the disparities of health care use [20]. Based on the literature, population density, per capita GCP, number of beds per 10,000 people, number of doctors per 10,000 people and number of nurses per 10,000 people in the county where the recipient lived were included as community-level enabling factors, which signify economic developments and medical resources in the counties that influence which health services are delivered [19]. Medical insurance schemes and Hukou status were not included in this study as more than 98% of the residents in these 73 counties were covered exclusively by the New Cooperative Medical Scheme and 95% of them were agricultural Hukou. More details about dependent and independent variables are presented in Table 2.

**Table 2:** Definitions of variables

Variables	
**Dependent variables**	
Length of the latest inpatient	The number of days for latest hospitalization in the past year
Number of admissions	The number of admissions during the previous year
Total inpatient expenditure	All the inpatient care cost in the past year (RMB)
Out-of-pocket (OOP) inpatient expenditure	All the self-paid inpatient care costs in the past year (RMB)
**Independent variables**	
**Predisposing factors**	
Gender	Dummy variable: Female = 0; Male = 1
Age	Categorical variables: If age less than 15 = 1; Otherwise = 0; If age 15–44 = 1; Otherwise = 0; If age 45–59 = 1; Otherwise = 0; If age older than 59 = 1; Otherwise = 0
Marital status	Dummy variable: Married = 0; Otherwise (Single, divorced or widowed) = 1
Education level	Categorical variable: Primary school or below = 1; Junior school = 2; Above junior school = 3
**Need factors**	
Chronic disease	Dummy variable: Whether diagnosed with chronic disease (e.g. hypertension); No chronic disease = 0; Chronic disease = 1
Health status ^a^	Categorical variable: the primary self-reported health status in the MFA data were: healthy, in good shape, generally, weak, disabled, seriously ill, and disabled with severe diseases. We recategorized the variable as: Very good or good = 1; Modest = 2; Disability or seriously ill = 3
**Enabling factors**	
Economic status	Categorical variable: Annual household income, grouped into quintiles; Quintile 1 (The poorest) = 1 –Quintile 5 (The richest) = 5
Time to the hospital	Categorical variable: Drive from residential districts to the nearest secondary/tertiary hospital (Minutes), were grouped into five groups; The shortest (time < 30) = 1; The shorter (30 < = time < 60) = 2; The medium (60 < = time < 90) = 3; The longer (90 < = time < 120) = 4; The longest (time > 120) = 5
Hospital grade	Dummy variable: The grade of medical institution where recipient’s latest inpatient care services were taken during the prior year; Secondary hospital = 0; Tertiary hospital =1
Population density	The number of individuals per unit geographic area where recipient lived (Person/km2)
Per capita GCP	GCP (Gross County Product) per capita of the county where recipient lived, calculated by dividing the GCP of a county by its population (Ten thousand yuan)
Number of beds per 10,000 people	Number of beds per 10,000 people of the county where recipient lived
Number of doctors per 10,000 people	Number of doctors per 10,000 people of the county where recipient lived
Number of nurses per 10,000 people	Number of nurses per 10,000 people of the county where recipient lived

Notes: ^a^ Given the unique of MFA population, they were generally poorer in health and those with disability and seriously illnesses accounted for a noticeable proportion of this population, self-reported health status in our study was not formulated as “excellent, very good, good, fair, and poor” or “very good, good, moderate, bad, and very bad”, instead, the item of “disability or seriously ill” was listed together as an alternative of “bad or very bad”

#### Multilevel model

A multilevel model was adopted to allow for the clustered data structure [30]. Here, recipients (individual-level) were nested within counties (community-level), thus a two-level linear mixed model was adopted to simultaneously estimate individual-level and community-level effects with the estimating equation:

$$ {y}_{ij}={\alpha}_0+{\alpha}_1{\mathcal{x}}_{ij}+{\alpha}_2{\mathcal{w}}_j+{\mathcal{u}}_j+{\varepsilon}_{ij} $$

In the specification, *y*_*ij*_ represents the inpatient care utilization of recipient *i* in county *j* and *α*_0_ means the intercept. ${\mathcal{x}}_{ij}$ and ${\mathcal{w}}_j$ are the individual-level and community-level variables, with corresponding coefficients of *α*_1_ and *α*_2_, respectively. ${\mathcal{u}}_j$ is the individual level error term while *ε*_*ij*_ denotes the community level error. In using a multilevel model, the intra-class correlation coefficient (ICC) of between-county heterogeneity in the community level needs to be statistically significant.

#### Moderation effect

Moderation effects, if significant, change the direction or magnitude of the association between independent and dependent variables [31]. In this study, guided by previous literature [22, 24, 25], we hypothesize that hospital grade would function as a moderator in the relationship between geographic access and inpatient care utilization in the MFA population. In order to test this moderating effect, hierarchical multiple regression was conducted. In this model, the occurrence of moderation can be observed by the significance of predictor and moderating variables as well as the general model R^2^ in Block 1 (without interaction term), and a significant interaction term and a significant R^2^ change in Block 2 (interaction term added). Adjusted predictions (marginal effect) of the interaction term were calculated for ease of interpretation of the interaction effect.

Descriptive statistics for the total sample by hospital grade summarize the characteristics of the MFA recipients. Mean (SD) and T-test were used for continuous variables, N (%) and Chi-square test were used for categorical variables. All statistical analyses were carried out using Stata, version 15.0.

## Results

Table 3 provides descriptive statistics for our study sample. Of the 9516 MFA recipients within 73 counties, more than half of the recipients were males (53.8%), aged above 45 years (75.8%), married (78.7%), and had an education level of primary school or below (86.6%). Regarding health status, 21.4% of recipients had chronic diseases and 32.3% reported having a disability or serious illness. The average driving time was 88.95 min from home to the closest hospital. In terms of inpatient care use, the average length of stay for the latest inpatient care was 21.1 days, and on average individuals sought inpatient treatment 1.38 times in the previous year, with a total annual inpatient expenditure of 20,828.01 RMB and an average OOP expense of 4954.13 RMB.

**Table 3:** Basic characteristics of variables for the total sample and comparisons between hospital grade

Variables	Total sample (*N* = 9516)	Hospital grade Tertiary hospital		*P* value
		Secondary hospital (*N* = 3592)	Tertiary hospital (*N* = 5924)	
Dependent variables				
Length of the latest inpatient, Mean (SD)	21.12 ± 36.38	18.32 ± 31.33	22.83 ± 39.03	†
Number of admissions, Mean (SD)	1.38 ± 1.00	1.47 ± 1.23	1.32 ± 0.83	†
Total inpatient expenditure, Mean (SD)	20,828.01 ± 32,884.50	10,446.77 ± 16,698.37	27,122.64 ± 38,251.28	†
OOP inpatient expenditure, Mean (SD)	4954.13 ± 11,954.08	1889.92 ± 5375.00	6812.11 ± 14,244.26	†
Independent variables				
Gender, N (%)				ns
Female	4394 (46.17)	1622 (45.16)	2772 (46.79)	
Male	5122 (53.83)	1970 (54.84)	3152 (53.21)	
Age, N (%)				†
< 15	371 (3.90)	90 (2.51)	281 (4.74)	
15–44	1937 (20.36)	628 (17.48)	1309 (22.10)	
45–59	3375 (35.47)	1198 (33.35)	2177 (36.75)	
> 59	3833 (40.28)	1676 (46.66)	2157 (36.41)	
Marital status, N (%)				ns
Married	7488 (78.69)	2837 (78.98)	4651 (78.51)	
Others	2028 (21.31)	755 (21.02)	1273 (21.49)	
Degree of education, N (%)				ns
Primary school or below	8179 (86.64)	3068 (86.06)	5111 (87.00)	
Junior school	1089 (11.54)	438 (12.29)	651 (11.08)	
Above junior school	172 (1.82)	59 (1.65)	113 (1.92)	
Chronic disease, N (%)				†
No chronic disease	7478 (78.58)	2680 (74.61)	4798 (80.99)	
Chronic disease	2038 (21.42)	912 (25.39)	1126 (19.01)	
Health status, N (%)				***
Very good or good	3222 (33.87)	1169 (32.54)	2053 (34.67)	
Modest	3217 (33.82)	1287 (35.83)	1930 (32.60)	
Disability or seriously ill	3074 (32.31)	1136 (31.63)	1938 (32.73)	
Economic status, N (%) ^a^				ns
Quintile 1 (The poorest)	1954 (20.55)	741 (20.63)	1213 (20.50)	
Quintile 2 (The poorer)	1893 (19.91)	682 (18.99)	1211 (20.47)	
Quintile 3 (The middle)	1879 (19.76)	752 (20.94)	1127 (19.05)	
Quintile 4 (The richer)	1907 (20.06)	711 (19.80)	1196 (20.21)	
Quintile 5 (The richest)	1875 (19.72)	705 (19.63)	1170 (19.77)	
Time to the hospital, N (%) ^b^				†
The shortest (time < 30)	2085 (21.91)	1817 (50.58)	268 (4.52)	
The shorter (30 < = time < 60)	1678 (17.63)	1119 (31.15)	559 (9.44)	
The medium (60 < = time < 90)	1859 (19.54)	381 (10.61)	1478 (24.95)	
The longer (90 < = time < 120)	1546 (16.25)	191 (5.32)	1355 (22.87)	
The longest (time > 120)	2348 (24.67)	84 (2.34)	2264 (38.22)	
Population density, Mean (SD)	296.53 ± 216.37	362.23 ± 228.81	256.69 ± 198.14	†
Per capita GCP, Mean (SD)	3.38 ± 2.22	3.68 ± 2.62	3.20 ± 1.92	†
Number of beds per 10,000 people, Mean (SD)	2.31 ± 0.68	2.33 ± 0.64	2.30 ± 0.71	*
Number of doctors per 10,000 people, Mean (SD)	0.61 ± 0.18	0.62 ± 0.18	0.59 ± 0.18	†
Number of nurses per 10,000 people, Mean (SD)	1.03 ± 0.33	1.06 ± 0.37	1.00 ± 0.30	†
Total Counties, N	73			

Notes: *p* values were calculated by hospital grades for recipients’ latest inpatient care. Mean (SD) and T-test were conducted for continuous variables; N (%) and Chi-square test were conducted for categorical variables. * *p* < 0.1, ** *p* < 0.05, *** *p* < 0.01, † *p* < 0.001. ns = not significant

^a^ The mean (SD) of annual household income is 8626.59 ± 5357.45 RMB

^b^ The mean (SD) of driving time is 88.95 ± 71.08 min to the nearest county/city level hospital, 38.48 ± 28.26 min to the secondary hospital and 119.56 ± 71.78 min to the tertiary hospital

*OOP* out-of-pocket

Among the study sample, 37.8% (3592) of recipients had their latest inpatient care in secondary hospitals whilst 62.3% (5924) were in tertiary hospitals. For those hospitalized in secondary hospitals, more than 80% of them required up to 60 min to drive to the nearest hospital, whereas only 13% were within a 1 h drive to the nearest tertiary hospital. When comparing those who were hospitalized in secondary hospitals and tertiary hospitals, significant differences were found in a range of characteristics, such as age, whether they had a chronic disease, health status, driving time to the hospital, as well as population density, per capita GCP, number of beds per 10,000 people, number of doctors per 10,000 people and number of nurses per 10,000 people for the counties where the recipient lived.

The ICC showed that 11.6% of the total variance in length of the latest inpatient stay (19.1% of the total variation in total inpatient expenditures and 10.4% of the total variance in self-paid inpatient expenditures, respectively) could be explained by community-level differences (for details see Appendix Table 1). On the other hand, for the number of admissions, only 4.6% of the total variance can be attributed to between-county differences, and therefore the OLS regression was used.

Table 4 shows the detailed regression results. After controlling for a wide range of confounding factors, we find that drive time to the latest hospital is significantly associated with inpatient care use. More specifically, compared to those with shortest drive time, all other drive time groups had significantly longer inpatient stays for the latest inpatient care, but fewer admissions (not significant for the medium and the longer drive time groups), and less annual total and OOP costs (not significant for the longest drive time group). Furthermore, compared to those admitted in secondary hospitals, recipients hospitalized in tertiary hospitals had a significantly shorter length of inpatient stay (β = − 3.971, *P* < 0.001), but higher total hospitalization expenses (β = 12,705.810, *P* < 0.001) and OOP hospitalization expenses (β = 4174.214, *P* < 0.001). These results indicate that for recipients who had inpatient care in tertiary hospitals, on average their inpatient stay was nearly 4 days shorter but total inpatient costs and OOP inpatient costs were 12,705.810 RMB and 4174.214 RMB higher, respectively, compared to their counterparts in secondary hospitals. Recipients’ characteristics, such as age, chronic disease, and population density of counties where recipients lived also had significant effects on all outcome variables of interest.

**Table 4:** Multilevel model analysis on influencing factors for inpatient health services utilization among the MFA recipients

Parameter	Length of the latest inpatient stay		Number of admissions last year		Total inpatient expenditure		OOP inpatient expenditure	
	Coef.	S.E.	Coef.	S.E.	Coef.	S.E.	Coef.	S.E.
Gender (Baseline: Female)								
Male	−0.059	0.715	0.022	0.020	1039.657*	602.581	298.069	232.507
Age (Baseline: Less than 15)								
15–44	−3.912*	2.103	0.200***	0.059	6930.009†	1771.072	1669.382**	683.310
45–59	−6.648**	2.153	0.122*	0.060	4631.393**	1813.155	649.546	699.516
> 59	−10.578†	2.152	−0.007	0.060	425.114	1812.229	− 475.304	699.118
Marital status (Baseline: Married)								
Others (Single, divorced or widowed)	−0.120	0.983	−0.010	0.027	− 2123.486**	828.287	− 730.073**	319.410
Degree of education (Baseline: Primary or below)								
Junior school	−0.101	1.154	0.059**	0.032	610.865	972.258	289.852	375.042
Above junior school	−3.830	2.709	0.600†	0.076	2377.716	2282.027	477.120	880.554
Chronic disease (Baseline: Chronic disease)								
No chronic disease	−2.201*	0.879	−0.084***	0.024	− 3134.205†	740.675	− 982.338†	285.784
Health status (Baseline: Very good or good)								
Modest	−0.487	0.901	−0.001	0.025	− 1278.351*	758.261	− 312.711	292.520
Disability or seriously ill	2.640***	0.901	0.122†	0.025	1505.424*	759.311	333.837	292.707
Economic status (Baseline: The poorest)								
The poorer	−0.226	1.203	0.011	0.032	− 1465.221	1013.662	− 534.044	390.298
The middle	0.542	1.258	0.010	0.032	− 1480.870	1060.670	− 449.249	407.840
The richer	−0.043	1.307	0.182†	0.033	− 1725.214	1101.501	− 529.951	423.252
The richest	−0.834	1.357	0.243†	0.034	− 142.134	1143.861	−140.945	439.182
Time to the hospital (Baseline: Shortest)								
The shorter	2.953**	1.192	−0.129†	0.032	− 2638.399***	1003.905	−893.349**	386.903
The medium	5.240†	1.442	−0.005	0.038	− 3432.667***	1215.533	−983.197**	467.568
The longer	5.229***	1.574	−0.062	0.039	− 4294.960**	1327.719	− 1383.912***	509.651
The longest	7.404†	1.928	−0.126**	0.044	− 2873.070*	1629.085	− 843.539	620.811
Hospital grade (Baseline: Secondary hospital)								
Tertiary hospital	−3.971***	1.176	−0.029	0.028	12,705.810†	992.024	4174.214†	380.325
Population density	−0.018**	0.009	0.001†	0.000	−21.954***	8.425	−5.300**	2.496
Per capita GCP	−1.289*	0.698	0.011	0.007	− 1666.647**	639.061	−359.208*	186.240
Number of beds per 10,000 people	−1.202	2.932	0.124†	0.024	− 7414.986***	2680.948	− 2571.946***	783.829
Number of doctors per 10,000 people	−3.396	8.552	−0.042	0.087	5245.711	7788.874	3967.324*	2321.577
Number of nurses per 10,000 people	0.376	7.212	−0.114*	0.060	6836.155	6584.274	2022.544	1936.436
Intercept	45.283†	7.080	0.796†	0.082	35,818.760†	6436.780	7679.606†	1930.638

Notes: Coef. means coefficient. * *p* < 0.1, ** *p* < 0.05, *** *p* < 0.01, † *p* < 0.001

*OOP* out-of-pocket

**Fig. 1:**
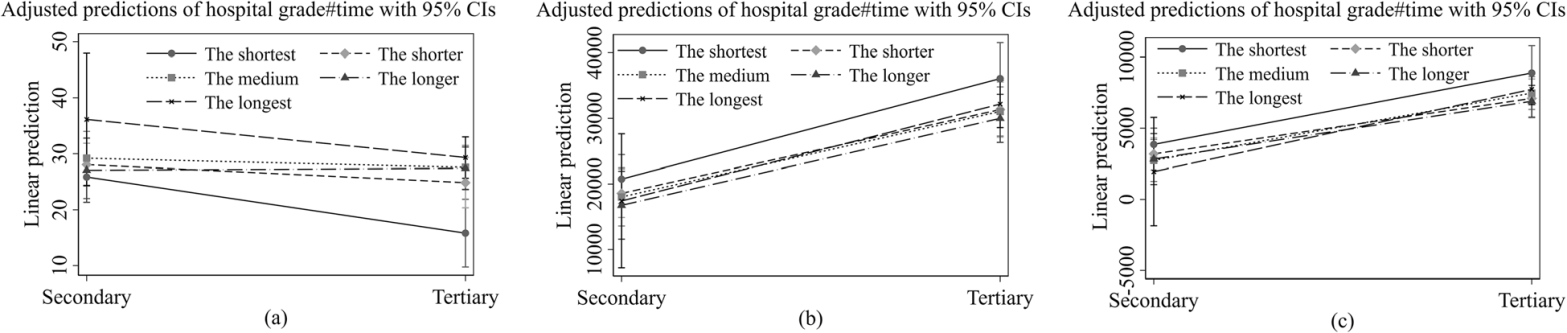
Adjusted predictions of the interaction terms on inpatient care use. **a** length of the latest inpatient stay, **b** total inpatient expenditure, and **c** OOP inpatient expenditure. Secondary means secondary hospital; Tertiary means tertiary hospital

We tested the moderation effect of hospital grade in the association between driving time to the hospital and inpatient care use by creating interaction terms between driving time to the hospital and the hospital grade. Significant R^2^ changes and interaction terms were observed in predicting length of the latest inpatient stay (Δ R^2^ = 0.012, *P* < 0.001), total inpatient cost (Δ R^2^ = 0.039, *P* < 0.001) and self-paid inpatient cost (Δ R^2^ = 0.032, *P* < 0.001). Detailed results for the hierarchical multiple regression examining the moderation effect are reported in Appendix Table 2, and the adjusted predictions of the interaction terms on inpatient care use are shown in Fig. 1.

Part (a) shows that no matter what time intervals the recipients were in, the length of inpatient stay was longer in secondary hospital than in a tertiary hospital: holding the other covariates at the mean level, the longest inpatient stay is predicted to be 36.1 days (*P* < 0.001) in a secondary hospital and 29.3 days (*P* < 0.001) in a tertiary hospital for the longest group. Part (b) shows that on average total inpatient expenditures in tertiary hospitals were higher than those in secondary hospitals and the maximum value of the marginal effect was 36,076.7 RMB (*P* < 0.001) in a tertiary hospital, which was observed in the shortest group. The above trend was also observed in predicating OOP inpatient expenses, with the maximum adjusted prediction of 8890.1 RMB (*P* < 0.001) in the tertiary hospital (Part (c)). These findings imply that regardless of the drive time, the total and OOP inpatient expenditures were higher in a tertiary hospital compared with a secondary hospital, and recipients who incurred the highest inpatient costs were those with the shortest time to the hospital.

The adjusted predictions of time to the hospital and hospital grade on inpatient services utilization in terms of remoteness are shown in Table 5. Compared to those with the shortest time to the hospital, when admitted in a secondary hospital, on average the length of inpatient stay in a tertiary hospital was significantly longer in the shorter (β = 9.035, *P* < 0.01), the medium (β = 11.769, *P* < 0.001), the longer (β = 11.581, *P* < 0.001) and the longest groups (β = 13.510, *P* < 0.001), respectively; whilst when admitted in a tertiary hospital, it was only significant in the shorter and the longest groups. After including the moderator variable, the impact of driving time to a hospital on length of the latest inpatient care was increased when admitted to a secondary hospital for all groups and in a tertiary hospital for the longest group. The above results suggest that the hospital grade enhanced the association between time to hospital and length of inpatient stay. Annual total and OOP inpatient expenditure were lower for recipients in the shorter, the medium, the longer and the longest than in the shortest group regardless of whether their treatments were obtained in a secondary hospital or a tertiary hospital. Moreover, the relationships between drive time to the hospital and both annual total and OOP inpatient costs were smaller when recipients were admitted to a tertiary hospital.

**Table 5:** Adjusted predictions of time to the hospital and hospital grade on inpatient services utilization

	Length of the latest inpatient stay				Total inpatient expenditure				OOP inpatient expenditure			
	Secondary hospital	Change ^b^	Tertiary hospital	Change ^c^	Secondary hospital	Change ^b^	Tertiary hospital	Change ^c^	Secondary hospital	Change ^b^	Tertiary hospital	Change ^c^
The shorter VS The shortest ^a^	9.035***	6.082	2.190*	−0.763	− 2167.083*	471.316	− 4735.390*	− 2096.991	−669.237	224.112	− 1790.303*	− 896.954
	(2.851)		(1.323)		(1126.240)		(2429.113)		(430.866)		(928.279)	
The medium VS The shortest ^a^	11.769†	6.529	3.336	−1.904	− 2717.816	714.851	− 5021.475**	− 1588.808	− 1121.277	−138.080	− 1416.147	−432.950
	(2.753)		(2.145)		(1829.125)		(2347.076)		(697.477)		(895.836)	
The longer VS The shortest ^a^	11.581†	6.352	1.200	−4.029	− 3991.857*	303.103	− 6073.508**	− 1778.548	− 1029.193	354.719	− 1992.204**	− 608.292
	(2.795)		(2.685)		(2288.859)		(2383.709)		(873.423)		(909.174)	
The longest VS The shortest ^a^	13.510†	6.106	10.269*	2.865	− 3320.336	− 447.266	− 3890.883	− 1017.813	− 1906.771	− 1063.232	− 1148.885	− 305.346
	(2.859)		(5.947)		(5071.255)		(2442.227)		(1934.536)		(928.394)	

Notes: ^a^ The Baseline. * *p* < 0.1, ** *p* < 0.05, *** *p* < 0.01, † *p* < 0.001

^b^ The change in coefficients when admitted to secondary hospital after moderation of hospital grade introduced; ^c^ The change in coefficients when admitted to tertiary hospital after moderation of hospital grade introduced

*OOP* out-of-pocket

## Discussion

This study investigated whether inpatient health services utilization varied by the remoteness, which was proxied by the drive time to the nearest hospital according to the actual driving time from Amap. The findings suggest that remoteness had a significantly negative effect on the frequency of inpatient care use in MFA recipients, which was further moderated by hospital grade.

Compared to those with the shortest drive time to the nearest hospital, the shorter, the medium, the longer and the longest had a significantly longer inpatient stay in the latest inpatient care but fewer admissions as well as lower total and OOP inpatient costs over the past year. Within the moderation of hospital grade, these links were increased in the relation between time to the hospital and length of the latest inpatient stay, while decreased when recipients were admitted to a tertiary hospital for both total and OOP inpatient expenditures. The reduction of inpatient admissions and annual total and OOP inpatient costs along with the increase in drive time reflects that the demand for health care reduced with the increase in travel distance. This geographic access barrier has played a significant role for whether to seek medical care and where to receive medical care when needed among MFA recipients. The unmet need may be an issue with the MFA population, however, further examination of this issue is beyond the capacity of our data [25, 32]. With the moderation of hospital grade, the relation between time to the hospital and inpatient costs were decreased, suggesting that even though there was a relatively lower frequency for recipients with longer driving time to the hospital, once they were admitted in a tertiary hospital, the total and OOP inpatient care costs increased regardless of drive time.

Longer drive time was associated with a longer (latest) inpatient stay. This finding is similar to a previous study for rural China, which reported that driving longer to the nearest clinic predicted a higher level of health service utilization in mountainous areas [10]. It might be that MFA recipients who live in remote mountainous and plateau areas generally have poor health status (eg. high-altitude areas expose humans to sustained hypoxia, which may lead to severe health problems) [10, 15]. Although the frequency of inpatient care was lower on average for residents living in far-off areas with poor roads and transportation, when their health status was worse (i.e. suffering severe illnesses or undergoing particular procedures), drive time tends to matter less. Therefore, when these residents decide to visit a doctor, they drive farther in pursuit of optimal medical treatment in faraway secondary or tertiary hospitals, given that high-quality care is concentrated in large counties or cities [15, 16, 32, 33]. This could be the reason that on average a single hospital stay is longer but the total number of hospitalizations and costs within a year are lower for recipients living in the low accessibility areas.

The results also indicated that hospital grade was significantly associated with recipients’ inpatient care use: the higher the level of hospital, the higher total and OOP inpatient expenses whilst the shorter length of the last inpatient stay. Higher total and OOP costs in tertiary hospitals are generally as expected [15, 25, 34, 35]. A plausible explanation for the shorter length of inpatient stay in a tertiary hospital is that tertiary hospitals have strict controls over the length of inpatient stays with high turnover rates of beds as more granular costing can be realized by amortizing their costs over a large volume of new patients, while secondary hospitals predominantly provide rehabilitation and nursing services and thus have relatively low turnover rates (according to China Health and Family Planning Statistical Yearbook 2017, the turnover rate of bed was 98.85 in tertiary hospitals and 84.1% in secondary hospitals in 2016) [36–38].

Considering other factors that influenced inpatients health utilization, as expected, compared to those subjectively perceived in good or very good health, recipients who reported disability or were seriously ill had longer inpatient stays as well as higher total and OOP inpatient expenses. Apart from individual factors, the community level characteristics also played a vital role in predicting recipients’ inpatient care use. Our study revealed that the increase in total beds per 10,000 people, population density and per capita GCP were related to a lower inpatient cost. It could be that residents in these counties with better health resources are generally wealthier and have better health status compared with those in less affluent areas.

Results are subject to a number of limitations. Firstly, only “Dibao” recipients that received ‘after medical treatment’ assistance were included from the MFA data. Ideally, we would like such data for all MFA recipients, however, hospitalization details of certain subgroups were incomplete in the available data and can’t be collected *ex*-post, which leads to other problems in assessing inpatient care utilization. For our purpose, the “Dibao” recipient is a more effective choice. Secondly, it is very common in the geographic access literature to use the village location as a proxy for the individual’s residence in rural areas as we have done. However, the drive time might be *under*-estimated. Further research would be benefit from taking each respondent’s residence as the minimum unit with more precise estimation. Thirdly, although the “diagnosis group for hospital admission” was not collected in the MFA data, other health need factors such as chronic disease and health status were adjusted in this study, enabling us to obtain a fine-tuned understanding of the inpatient health services utilization of MFA recipients. Finally, since only cross-sectional data for MFA recipients was obtained in our analysis, the associations we observed should not be interpreted as causal.

Despite these limitations, to the best of our knowledge, this study is one of only a few efforts to assess geographic access based on digital maps with real-time transportation information, thus offering higher precision of geographic access in MFA recipients in a developing country; it is also the first investigation of this topic concentrating on the MFA recipients in China. Our findings suggest further development of MFA program to offer more assistance to those in remote areas would avoid potential barriers to timely and adequate care. The benefit package should also cover the indirect medical costs such as transportation costs for the most vulnerable recipients.

## Conclusion

Our results suggest that time to the hospital and hospital grade are salient predictors of inpatient care use, and there is a significant moderation effect of hospital grade. Compared to those with the shortest drive time to the nearest hospital, recipients with longer drive time had significantly lower frequency of inpatient care use with the most distant residents most affected. These findings provide novel evidence of the weak access for the extremely poor and unique insights for pro-poor health strategies as well as further development of MFA scheme.

## Supplementary Information

**Additional file 1: Appendix Table 1.** Two level null model on inpatient services utilization of MFA recipients. **Appendix Table 2.** Test results of hierarchical multiple regression in examining moderation effect.

## Data Availability

Data used in this study belongs to the Surveillance System of Civil Affairs of Shaanxi province and contains personal information (e.g., name, ID, etc.) of recipients. Due to the sensitive nature of these data and restrictions imposed by the institution, the authors cannot make these data publicly available. Other researchers who want to use the data may contact the author for data requests.

## Abbreviations

MFA: Medical Financial Assistance
CHE: Catastrophic Health Expenditure
OLS: Ordinary Least Squares
GCP: Gross County Product
OOP: Out-of-pocket
ICC: Intra-class Correlation Coefficient

## References

[1] Yip W, Hsiao W, Chen W (2012). Early appraisal of China’s huge and complex health-care reforms. Lancet.

[2] Liu G, Vortherms S, Hong X (2017). China’s health reform update. Annu Rev Public Health.

[3] Li J (2019). Achievements and challenges of health poverty alleviation in China. Seeker.

[4] Hu R, Dong S, Zhao Y (2013). Assessing potential spatial accessibility of health services in rural China: a case study of Donghai County. Int J Equity Health.

[5] Liu M, Zhang Q, Lu M (2007). Rural and urban disparity in health services utilization in China. Med Care.

[6] Zimmer Z, Kwong J (2004). Socioeconomic status and health among older adults in rural and urban China. J Aging Health.

[7] Akin J, Dow W, Lance P (2005). Changes in access to health care in China, 1989–1997. Health Policy Plan.

[8] Gu X, Zhang L, Tao S (2019). Spatial accessibility to healthcare services in metropolitan suburbs: the case of Qingpu, Shanghai. Int J Environ Res Public Health.

[9] Shi W, Chongsuvivatwong V, Geater A (2011). Effect of household and village characteristics on financial catastrophe and impoverishment due to health care spending in Western and central rural China: a multilevel analysis. Health Res Policy Syst.

[10] Fang P, Han S, Zhao L (2014). What limits the utilization of health services among the rural population in the Dabie Mountains-evidence from Hubei province, China?. BMC Health Serv Res.

[11] Fang P, Su M (2017). A discussion on the key problems and system construction of health poverty alleviation in China. Chin J Health Policy.

[12] Liu K, Yang J, Lu C (2017). Is the medical financial assistance program an effective supplement to social health insurance for low-income households in China? A cross-sectional study. Int J Equity Health.

[13] Hai F. International health care system profiles. China; 2020. https://www.commonwealthfund.org/international-health-policy-center/countries/china. Accessed 5 June 2020.

[14] Ma X, Zhang J, Meessen B (2011). Social health assistance schemes: the case of medical financial assistance for the rural poor in four counties of China. Int J Equity Health.

[15] Qian D, Pong R, Yin A (2009). Determinants of health care demand in poor, rural China: the case of Gansu Province. Health Policy Plan.

[16] Han Y, Wei J, Song X (2012). Accessibility of primary health care workforce in rural China. Asia Pac J Public Health.

[17] Penchansky R, Thomas W (1981). The concept of access: definition and relationship to consumer satisfaction. Med Care.

[18] Derose K, Gresenz C, Ringel J (2011). Understanding disparities in health care access-and reducing them-through a focus on public health. Health Aff.

[19] Levesque J, Harris M, Russell G (2013). Patient-centred access to health care: conceptualising access at the interface of health systems and populations. Int J Equity Health.

[20] Aday L, Andersen R (1981). Equity of access to medical care: A conceptual and empirical overview. Med Care.

[21] Center for Health Statistics and Information. An Analysis Report of National Health Services Survey in China. Beijing: Beijing Union Medical University Press; 2008. (in Chinese).

[22] Cheng G, Zeng X, Duan L (2016). Spatial difference analysis for accessibility to high level hospitals based on travel time in Shenzhen, China. Habitat Int.

[23] Wu C, Fang P (2007). Analysis of inequity of health Service for Rural Residents in West China and potential accessibility and countermeasure. Chin Health Serv Manag.

[24] Li X, Huang J, Zhang H (2008). An analysis of hospital preparedness capacity for public health emergency in four regions of China: Beijing, Shandong, Guangxi, and Hainan. BMC Public Health.

[25] Yu W, Li M, Ye F, et al. Patient preference and choice of healthcare providers in Shanghai, China: a cross-sectional study. BMJ Open. 2017;7(10):2–11. 10.1136/bmjopen-2017-016418 PMC5695435 29092898

[26] Al-Taiar A, Clark A, Longenecker J (2010). Physical accessibility and utilization of health services in Yemen. Int J Health Geogr.

[27] Boscoe F, Henry K, Zdeb M (2012). A nationwide comparison of driving distance versus straight-line distance to hospitals. Prof Geogr.

[28] Quan B, Gang X, Yong, et al. (2014). Analysis and detection of bogus behavior in web crawler measurement. Procedia Comput Sci.

[29] Hsia R, Shen Y. Changes in geographical access to trauma centers for vulnerable populations in the United States. Health Affairs (Project Hope). 2011;30(10):1912. 10.1377/hlthaff.2011.0510 PMC3287365 21976335

[30] Israel S (2016). How social policies can improve financial accessibility of healthcare: a multi-level analysis of unmet medical need in European countries. Int J Equity Health.

[31] Bolin J, Hayes A. Introduction to mediation, moderation, and conditional process analysis: a regression-based approach. New York: The Guilford Press. J Educ Meas. 2014;51(3):201–45.

[32] Liu Y, Zhong L, Yuan S, et al. Why patients prefer high-level healthcare facilities: a qualitative study using focus groups in rural and urban China. BMJ Global Health. 2018;3(5):2–7. 10.1136/bmjgh-2018-000854 PMC6150133 30258653

[33] Chen R, Du X, Yang Z (2016). Analysis of choice of healthcare services and the influencing factors among outpatients in Chengdu city. Mod Prev Med.

[34] Meng Q, Mills A, Wang L (2019). What can we learn from China’s health system reform?. BMJ.

[35] Yip W, Wang H, Liu Y (1998). Determinants of patient choice of medical provider: a case study in rural China. Health Policy Plan.

[36] Ma W. Research on the checks and balances mechanism of medical insurance cost control. Tianjin: Tianjin University; 2010. (in Chinese).

[37] Taheri P, Butz D, Greenfield L (2000). Length of stay has minimal impact on the cost of hospital admission. J Am Coll Surg.

[38] Li F, Sang P, Zhu B (2019). Analysis of hospitalization behavior of death patients with malignant tumors in Shanghai. China Health Econ.

