# Supplementary material for: Does inpatient health services utilization vary by remoteness in the medical financial assistance population? Evidence from Shaanxi province, China
Source: BMC Health Serv Res. 2020 Nov 19;20:1051. doi: 10.1186/s12913-020-05907-x (PMC7678078; doi:10.1186/s12913-020-05907-x)
Supplement: Supplementary file 1 — Additional file 1: Appendix Table 1. Two level null model on inpatient services utilization of MFA recipients. Appendix Table 2. Test results of hierarchical multiple regression in examining moderation effect. [file 12913_2020_5907_MOESM1_ESM.pdf]

Appendix Table 1 Two level null model on inpatient services utilization of Medical Financial Assistance (MFA) recipients

Parameter	Length of the latest inpatient stay			Number of admissions last year			Total inpatient expenditure			OOP inpatient expenditure		
	Estimate	S.E.	95% CI	Estimate	S.E.	95% CI	Estimate	S.E.	95% CI	Estimate	S.E.	95% CI
ICC	0.116	0.021	(0.068, 0.151)	0.046	0.009	(0.025, 0.060)	0.191	0.030	(0.138, 0.256)	0.104	0.019	(0.072, 0.149)

Notes: * p<0.1, ** p<0.05, *** p<0.01, † p<0.001.

OOP, out-of-pocket.

Appendix Table 2 Test results of hierarchical multiple regression in examining moderation effect

	Length of the latest inpatient stay		Number of admissions last year		Total inpatient expenditure		OOP inpatient expenditure	
	Block 1	Block 2	Block 1	Block 2	Block 1	Block 2	Block 1	Block 2
Time to the hospital	0.007***	0.007*	-0.001**	-0.001**	21.153†	-49.661**	5.850***	-16.988**
Hospital grade	-1.035†	-1.040***	-0.015	-0.025	9670.597†	6349.685†	3167.962†	2096.963†
Time to the hospital # Hospital grade		-0.014***		0.001		74.462†		24.015***
R^2	0.414†	0.426†	0.453†	0.461†	0.359†	0.398†	0.357†	0.389†
ΔR^2		0.012†		0.008†		0.039†		0.032†

Notes: $\Delta R^2 = R^2_{Block2} - R^2_{Block1.}$

* *p*<0.1, ** *p*<0.05, *** *p*<0.01, † *p*<0.001.

OOP, out-of-pocket.
